# Cytoreductive Nephrectomy Following Immunotherapy-Base Treatment in Metastatic Renal Cell Carcinoma: A Case Series and Review of Current Literature

**DOI:** 10.3390/curroncol28030178

**Published:** 2021-05-20

**Authors:** Scott J. Dawsey, Steven C. Campbell, Moshe C. Ornstein

**Affiliations:** 1 Department of Hematology & Medical Oncology, Cleveland Clinic Taussig Cancer Institute, Cleveland, OH 44106, USA; dawseys@ccf.org; 2 Center for Urologic Oncology, Glickman Urologic & Kidney Institute, Cleveland Clinic, Cleveland, OH 44106, USA; campbes3@ccf.org

**Keywords:** metastatic renal cell carcinoma, immunotherapy, cytoreductive nephrectomy, case report

## Abstract

The role and timing of cytoreductive nephrectomy in patients with metastatic renal cell carcinoma receiving immunotherapy-based regimens is unclear. However, the ability to achieve a complete response for metastatic renal cell carcinoma likely requires a nephrectomy at some point during treatment. Here we present a case series of three patients with metastatic clear-cell renal-cell carcinoma who received front-line immunotherapy-based treatment and subsequently underwent a cytoreductive nephrectomy. All three patients had a complete response to therapy and have subsequently remained off systemic therapy for a median of 531 days (range, 476–602). We also review the limited literature in this setting and highlight ongoing clinical trials. Although the role of cytoreductive nephrectomy in patients with metastatic renal cell carcinoma receiving immunotherapy-based treatment is uncertain, a subset of patients will benefit from either an immediate or deferred cytoreductive nephrectomy. Ongoing trials are underway to further determine how to incorporate cytoreductive nephrectomy into the treatment paradigm for patients with metastatic renal cell carcinoma.

## 1. Introduction

Historically, an overall survival (OS) benefit was demonstrated for cytoreductive nephrectomy (CN) followed by interferon-α (INF-α) for the treatment of metastatic renal cell carcinoma (mRCC) [1,2]. In the Tyrosine Kinase Inhibitor (TKI) era of treatment for mRCC, the role and timing of CN became less clear and guidelines were based primarily on retrospective studies [3]. Subsequently, two prospective trials (SURTIME and CARMENA) were conducted, which highlighted that CN was not appropriate for all patients but that some patients still benefit from an immediate or delayed CN [4,5]. 

The above data are limited to patients receiving targeted therapies (e.g., sunitinib) as treatment for mRCC. However, more recently, the standard of care for treatment of mRCC has shifted and the backbone of therapy is immunotherapy (IO). IO-based combinations such as axitinib plus avelumab [6], axitinib plus pembrolizumab [7], cabozantinib plus nivolumab [8], ipilimumab plus nivolumab [9], and lenvatinib plus pembrolizumab (pending FDA approval) [10] are now the foundation of therapy for patients with mRCC. Prospective data for CN in patients receiving these therapies are limited. In extrapolations from prior trials, factors such as the presence of systemic symptoms, burden of extra-renal disease, and International Metastatic RCC Database Consortium (IMDC) risk can potentially guide the decision for CN in mRCC in this setting [11]. However, the role and timing of CN after treatment with IO-based therapy remains uncertain. 

It does appear that a CN at some point over the course of mRCC treatment is critical in achieving a complete response (CR) [12]. A CR is important to achieve an extended treatment-free interval and durable response for patients with mRCC. Herein, we present three cases of CN following IO-based treatment in mRCC, review the current literature in this setting, and highlight ongoing clinical trials.

## 2. Case Presentation

### 2.1. Case 1

A 50-year-old man was diagnosed with metastatic clear-cell renal-cell carcinoma (mccRCC) without variant histology. The primary left renal tumor measured 13.5 cm × 11.6 cm. The patient had metastases to bilateral lungs and cervical spine. There was a 57-day interval from diagnosis to initiation of systemic treatment. Karnofsky Performance Status (KPS) was 90%. Laboratory testing demonstrated hemoglobin (Hgb), 12.5 g/dL; absolute neutrophil count (ANC), 4.16 k/µL; platelet (Plt) count, 349 k/ µL; calcium (Ca), 9.7 mg/dL. IMDC score was 1. The patient received four cycles of combination therapy with ipilimumab 1 mg/kg/dose plus nivolumab 240 mg/dose, followed by 10 cycles of single agent nivolumab 480 mg/dose every 4 weeks. The best treatment response was stable disease (SD), based on RECIST 1.1, achieved after 133 days of treatment. The patient underwent a left CN 15 days after his final dose of IO as he showed a significant improvement in pulmonary nodules, no evidence of recurrence of cervical spine lesion status post laminectomy, and stable left renal disease. Post CN, the patient remains off therapy with a 476-day treatment-free interval (TFI), during which time the remaining lung nodules have remained stable.

### 2.2. Case 2

A 61-year-old man was diagnosed with mccRCC without variant histology. The primary left renal tumor measured 10 cm in the largest dimension. The patient had metastases to a right hilar lymph node, left acetabulum, and posterior ischium. Given the patient’s pain, he underwent radiation to the left pelvis. There was a 48-day interval from diagnosis to initiation of systemic therapy. KPS was 100%. Laboratory testing demonstrated Hgb, 13.4 g/dL; ANC, 5.33 k/µL; Plt count, 253 k/µL; Ca, 9.2 mg/dL. IMDC risk score was 1. The patient received 3 cycles of combination therapy with ipilimumab 1 mg/kg/dose plus nivolumab 240 mg/dose. The best treatment response was partial response (PR), based on RECIST 1.1, achieved after 77 days of treatment. IO was discontinued due to 5 days of fevers with negative infectious workup. The patient underwent a left CN 126 days after his final dose of IO, as he showed improvement in all sites of disease. Post CN, he remains off therapy with a 531-day TFI, during which time adenopathy and bone lesions have remained stable. 

### 2.3. Case 3

A 58-year-old man was diagnosed with mccRCC without variant histology. The primary left renal tumor measured 4.6 cm × 6.4 cm. The patient had metastases to lung, left clavicle, and L5 vertebrae. There was a 76-day interval from diagnosis to initiation of systemic treatment. At treatment initiation, KPS was 80%. Laboratory testing demonstrated Hgb, 13.1 g/dL; ANC, 3.11 k/µL; Plt count, 184 k/ µL; Ca, 9.4 mg/dL. IMDC risk score was 1. The patient received combination therapy with nivolumab plus sunitinib (on clinical trial) for six years. The best treatment response was PR, based on RECIST 1.1, achieved after 671 days of treatment. The patient stopped IO and TKI due to progression of renal lesion. The patient underwent a left CN 59 days after last IO and 18 days after his final dose of TKI as he had progression of renal mass. Post CN, the patient has had a 602-day TFI (Table 1), during which time lung and bone lesions have remained stable.

## 3. Discussion

We have presented three cases of patients with mRCC who received IO-based combination therapy followed by CN and remained off systemic therapy for a median of 531 days (range, 476–602). This TFI may be biologically explained by CN causing diminished tumor mediated immunomodulation [13,14,15,16,17] and nephrectomy-induced azotemia [18]. The role of CN in patients with mRCC receiving IO-based therapy is unclear. We searched pubmed and recent oncology conferences for case series on post-IO nephrectomies in mRCC and pertinent clinical trials. Two recent clinical trials in patients receiving targeted therapy with sunitinib have demonstrated that although a CN should not be the standard of care for all patients, there is a subset of patients that will benefit from a CN.

The CARMENA trial was a phase III non-inferiority trial comparing CN followed by sunitinib, versus sunitinib alone in patients with mRCC [5]. Patients were randomized by their Memorial Sloan Kettering Cancer Center prognostic model score. CN was performed within 28 days of randomization and then sunitinib was then initiated 3–6 weeks after CN, whereas in the sunitinib-alone group, sunitinib was initiated within 21 days of randomization. Initial sunitinib dosing of 50 mg daily in cycles of 28 days on followed by 14 days off every 6 weeks, and dose modifications were allowed as needed to manage adverse events. The primary endpoint was OS, defined as the time from randomization until death from any cause or until the date of last contact for living patients. The median follow up was 50.9 months. The median OS for the sunitinib alone group was 18.4 months (95% CI, 14.7 to 23.0) versus 13.9 months (95% CI, 11.8 to 18.3) in the CN followed by sunitinib group. The hazard ratio for death in the analysis of OS was 0.89 (95% CI, 0.71 to 1.10; upper bound for non-inferiority was ≤ 1.20); thus, sunitinib was found to be non-inferior to CN followed by sunitinib [5]. 

The SURTIME trial was a phase III trial which randomized patients to upfront CN followed by sunitinib (“immediate CN”) versus upfront sunitinib followed by CN (“deferred CN”), and then additional sunitinib in patients with mRCC [4]. In the immediate CN group, sunitinib was started 4 weeks post CN, whereas in the deferred CN group, CN was performed after the third cycle of sunitinib and sunitinib was resumed 4 months post CN. Initial sunitinib dosing of 50 mg daily in cycles of 28 days on followed by 14 days off every 6 weeks, and dose modifications were allowed as needed to manage adverse events. 

Due to poor accrual, the study closed early and with a primary endpoint of a 28-week progression-free rate (PFR). A total of 99 patients were randomized and the median follow up was 3.3 years. The PFR at 28 weeks was 42% and 43% in the immediate vs. deferred CN arms, respectively (*p* = 0.61). In the intent-to-treat population, the OS favored deferred CN (HR 0.57; 95% CI, 0.34–0.95; *p* = 0.03) but this finding was not statistically significant in the per-protocol population (HR 0.71; 95% CI, 0.40–1.24; *p* = 0.23) [4]. Taken together, these data suggest that an upfront CN should not be the standard of care for all patients with mRCC. However, as there was discordance between the intent-to-treat population and the per protocol population OS outcome, there is likely a subset of patients that benefit from systemic therapy followed by CN.

Recently, a number of case series and retrospective reports demonstrate the benefit of CN in a subset of patients receiving IO-therapy. In a retrospective review of 391 mRCC patients from the United States national cancer database from 2015 to 2016, patients who underwent CN plus IO had superior OS compared to those who received IO alone. Additionally, two of twenty patients who had IO followed by CN achieved a complete pathologic response of the primary tumor [19]. Similarly, a retrospective review of 3856 patients with de novo mRCC that included 143 patients who received IO followed by CN, and 55 who received IO without CN, a statistically significant OS benefit was found in the CN group (Hazard Ratio= 0.39 [0.19–0.83]) [20] There are additional case reports of complete responses (CR) in patients with mRCC who received second-line IO [19,21] or first-line IO plus IO [22]. Of note, in a case series of eleven patients with mRCC who received IO as first- or second-line therapy, followed by CN, to achieve CR, 6 of 11 patients were free from systemic therapy at 1 year [23]. Interestingly, CN in this case series was technically more difficult, due to inflammation obscuring surgical dissection planes.

There are two clinical trials underway to help guide the use of CN and IO in mRCC. The PROBE (NCT04510597) trial will evaluate the combination of CN followed by systemic therapy (IO alone or TKI + IO) for mRCC as opposed to systemic therapy alone with IO or TKI + IO [24]. The NORDIC-SUN (NCT03977571) trial will evaluate the role of deferred CN in patients receiving combination IO (Nivolumab + Ipilimumab) [25]. Finally, the CYTOSHRINK (NCT04090710) trial will evaluate the use of cytoreductive stereotactic body radiation therapy instead of CN in patients with mRCC who received IO [26].

## 4. Conclusions

The role of CN in patients with mRCC receiving immunotherapy-based treatment is uncertain. It is clear, however, that an undefined subset of patients will benefit from either an immediate or deferred CN, which may result in a CR or at least provide an extended TFI. Urgently needed clinical trials are underway to further determine how to incorporate CN into the treatment paradigm for the correct patients with mRCC. The identification of molecular biomarkers through future clinical trials will also be invaluable to further refine treatment planning. As we await level one evidence about the role of CN in IO-treated patients, those who have had a good response to treatment can be reviewed at multidisciplinary tumor boards for the consideration of CN on a case-by-case basis.

## Figures and Tables

**Table 1:** Patient Characteristics.

Patient	Age	Histology	IMDC	Treatment	Treatment Duration (Days)	Surgical Pathology CN	TFI (Days)
1	50	Clear Cell	1	Ipilimumab and Nivolumab followed by Nivolumab Maintenance	347	100% ccRCC, 13.5 cm, grade 3, 1% necrosis, pT3a	476
2	61	Clear Cell	1	Ipilimumab and Nivolumab	43	100% ccRcc, 5.5 cm, grade 3, 5% necrosis, pT1b	531
3	58	Clear Cell	1	TKI plus nivolumab	2205	100%ccRcc, rhabdoid features, 5.3 cm, grade 4, 30% necrosis, pT3a	602
All (Median)	56.3		1		865		536

## Data Availability

Data available on request due to privacy restrictions. The data presented in this study are available on request from the corresponding author. The data are not publicly available due to being part of the electronic medical record.
